# Substance use disorder treatment retention and completion: a prospective study of horse-assisted therapy (HAT) for young adults

**DOI:** 10.1186/s13722-015-0043-4

**Published:** 2015-10-14

**Authors:** Ann Kern-Godal, Espen Ajo Arnevik, Espen Walderhaug, Edle Ravndal

**Affiliations:** Department of Addiction Treatment, Oslo University Hospital, Sognsvannsveien 21, Building 22, 0424 Oslo, Norway; Department of Psychology, University of Oslo, Oslo, Norway; Norwegian Centre for Addiction Research (SERAF), University of Oslo, Oslo, Norway

**Keywords:** Addiction, Substance use, Treatment completion, Duration of treatment, Dropout, Horse-assisted therapy (HAT), Equine-assisted psychotherapy (EAP), Alternative/complementary treatment

## Abstract

**Background:**

Keeping substance use disorder patients actively engaged in treatment is a challenge. Horse-assisted therapy (HAT) is increasingly used as a complementary therapy, with claimed motivational and other benefits to physical and psychological health. This naturalistic study aimed to assess HAT’s impact on the duration and completion of treatment for young substance users at Oslo University Hospital.

**Methods:**

Discharge and other data were derived from the Youth Addiction Treatment Evaluation Project (YATEP) database for patients (n = 108) admitted during an 18-month period. An intention-to-treat design, and univariate and multivariate analyses were used to compare those receiving treatment as usual (n = 43) with those who received treatment as usual plus HAT (n = 65).

**Results:**

Despite a lack of randomization, the baseline characteristics of the two groups were similar. However, more HAT participants completed treatment (56.9 vs 14 %, p < 0.001), remained in treatment for longer (mean 141 vs 70 days, p < 0.001) and had a significantly higher chance of completing their treatment than those not given the HAT program. Excluding time in treatment, and after controlling for the potentially confounding influence of age, sex, education, number and severity of substances used, psychological distress and number of temporary exits, the adjusted odds ratio for treatment completion was 8.4 in the HAT group compared with those not participating in HAT (95 % CI 2.7–26.4, p < 0.001).

**Conclusion:**

The study found a statistically significant association between HAT participation and time in treatment, and between HAT participation and completion of treatment. This association does not infer causality. However, it adds supporting evidence for the development of an innovative therapy, and warrants investment in further research in relation to its inclusion in substance use disorder treatment.

## Background

Retention in treatment improves the prognosis for substance use disorder patients [1–3]. Early dropouts are reported to have the same outcome as untreated patients [3]. There have been four major reviews of dropout from addiction and substance use disorder treatment [3–6], and these involve more than 500 studies undertaken over almost 40 years. They report that despite wide diversity in treatment methods [7], patient failure to complete therapy (usually referred to as dropout) often exceeds 50 %. Completion of treatment is associated with successful outcomes [3, 4, 8, 9]. The optimal duration of treatment is debatable and may depend upon the treatment method [10], but 90 days is often identified as the minimum time period for effective treatment [2, 3, 11–13]. In addition, many substance use patients exit treatment for various reasons and then re-enter treatment after varying periods of absence [3, 4, 13]. There is a continuous struggle to find treatment modalities that motivate patients to remain for sufficient time to enable beneficial change in morbidity [10]. Treatment factors such as method, staff–patient alliance and interaction, and satisfaction, although less frequently studied, have been found to be among the best predictors of outcome [6]. Reported studies of alternative or complementary treatment methods are rare [14], including those for substance use disorders.

Horse (or equine)-assisted/facilitated therapy is an innovative complementary approach to psychotherapy that actively involves horses or other equines in the therapeutic process. Challenges in this rapidly developing field of experimental therapy include increasing the provision of a high-cost therapy, which is often of unknown quality, to vulnerable population groups with little substantiating evidence of associated benefits [15].

Since the 1990s, there has been a dramatic increase in the number of equine programs that claim to provide psychotherapy and/or education and development. However, equine-assisted/facilitated psychotherapy is still very much in an evolutionary phase, lacking a generally accepted theoretical framework. There is a variety of emerging schools of thought, approaches and terminology [16–18]. The term horse-assisted therapy (HAT) is used in this paper.

A review of the literature and media relating to psychotherapy involving horses has revealed a growing number of studies and “opinion-based” material in the psychosocial area with a variety of factors at play, including motivation. These include claims that the size, strength, warmth, body language and herd behavior of horses can be used with therapeutic benefit when working with clients who are mistrusting, depressed and anxious [16, 18–21], or who lack the boundary setting or other skills needed to deal with everyday living [22, 23] or who have issues related to self-esteem, self-efficacy or resilience [24, 25]. In addition, there are claims that the horse “mirrors” the patient and provides immediate, honest feedback, untainted by the usual human and social constraints [16, 21], and that the horse can promote trust in vulnerable clients, particularly those with traumatic backgrounds [20, 26]. Dell and colleagues refer to the importance of the horse’s consistency, and nonverbal and nonjudgmental relationship with Inuit youth undergoing substance abuse treatment [27]. In general, the horse is reported to be a motivational force for treatment [28–30].

However, the studies are usually small and rarely documented in an adequate or systematic format. There have been two published systematic reviews of peer-reviewed literature of psychotherapeutic programs involving horses [15, 31]. The first reviewed material in 16 databases, identifying 103 studies, of which 14 met the selection criteria. Only two of these were rated as having evidence of (moderate) effectiveness [31]. The second, in mid-2014, reviewed 14 studies identified from a more restricted search and found that all 14 were compromised by threats to validity. The authors concluded that psychotherapy involving equines should not be marketed but that research should continue with improved methodology [15]. Since then, findings from a randomized controlled study have been published. The study found that long-term psychiatric patients at risk of violence responded positively to a program of equine-assisted psychotherapy [32].

Many horse centers offer therapy for substance use, or addiction programs [33], but few studies are reported. We found nine specific HAT and substance use disorder-related papers or theses, only one of which was in a peer-reviewed journal [27]. None of them met the inclusion criteria for the two systematic reviews.

Most reported studies of HAT, including those relating to substance use and addiction, conclude with a recommendation for further research. However, few HAT programs have the resources, patient numbers, diagnostic homogeneity, or the research capacity and skills required.

In 2010, Oslo University Hospital’s Department of Addiction Treatment—Youth provided a unique research opportunity to study HAT. It had approximately 100 new patients per year with a primary diagnosis of substance use and/or addiction. In addition, it had 37 years’ experience of HAT in a residential psychiatric setting [22]. Since 2010, the hospital’s resident herd of five specially selected and trained horses has worked exclusively with the department’s young patients in a structured, substance use disorder-relevant program of HAT.

As far as we are aware, this is the first peer-reviewed quantitative study of the inclusion of HAT in a substance use disorder treatment program. Our objective was to assess whether HAT patients remained in treatment longer and were more likely to complete their agreed program of treatment. We hypothesized that HAT participation was associated with both longer time in treatment and completion of treatment.

## Methods

The study covered an 18-month treatment period from January 1, 2011. It was part of a larger, ongoing, mixed-methods project to investigate the impact of HAT on substance use disorder treatment outcomes.

Patient participation was voluntary. All necessary patient consent and data inspection authority approvals were obtained as part of the Youth Addiction Treatment Evaluation Project (YATEP). The study was reviewed and approved by the Norwegian Regional Committee for Medical Research Ethics, and performed according to their guidelines and the Helsinki Declaration.

### Patients

The study sample comprised inpatients and day patients admitted between January 1, 2011 and June 30, 2012 to the Department of Addiction Treatment—Youth at Oslo University Hospital. The department treats men and women aged 16–26 years (but patients up to 35 years of age may be accepted) who have a primary diagnosis of mental and behavioral disorders due to psychoactive substance use (ICD 10). One hundred eleven patients entered treatment during the 18-month period. Three patients were discharged to other institutions for ongoing treatment, leaving 108 patients in the study.

### Study design

At entry to treatment, all participating patients who had provided written informed consent were registered in the YATEP database. Recording usually started in the first week. The database comprises basic patient information, psychological tests, discharge status, and HAT participation data. Additional patient demographic, morbidity and treatment information, plus the dates of any temporary exit from treatment, can be drawn from the hospital’s electronic patient journal when required and matched anonymously to the YATEP database records. All individuals were followed from treatment entry to discharge.

### Measures

The study outcomes were: (1) completion of treatment (primary outcome), (2) time in treatment (measured using number of treatment days), and (3) completion of 90 days of treatment or more (included because it is often identified as a critical period for effective treatment [2, 3, 11–13]).

Discharge status was categorized as (1) treatment completed, or (2) dropout. Treatment completed was defined as staying in treatment for the duration of the recommended treatment plan. This was determined by examining the YATEP discharge report and the clinician’s journal record. Those who left the program but returned within a 30 day period to continue with the remainder of the treatment course were considered to have completed treatment. Leaving the program but returning within a 30 day period was termed “temporary exit.” The number of days of temporary exit was excluded from the total treatment days at discharge. Dropout was defined as patient initiated treatment termination, or expulsion for rule violation prior to completing the agreed treatment period.

Psychological distress was measured using the Hopkins Symptom Check List 25 (HSCL-25), which is one of the assessment items in the YATEP. It consists of 25 questions that map respondents’ anxiety and depression [34]. It is scored on a scale from 1 (not bothered) to 4 (extremely bothered). The form is frequently used in Norwegian research projects with 1.75 as the risk cutoff in normal Norwegian populations [35]. In the analysis for this study, HSCL-25 was used as an indicator of psychological distress.

Severity of substances used was categorized as more severe [heroin, amphetamine, benzodiazepine, gamma hydroxybutyrate (or GHB) and cocaine] or less severe (cannabis and alcohol).

### Treatment as usual (TAU)

The treatment site is part of the specialist health care system in Norway. Patients are referred by general practitioners and specialists or from other hospital departments. They must have a primary diagnosis of mental and behavioral disorders due to psychoactive substance use (ICD 10). The social services authority has oversight of this process. The treatment is a person-centered program which comprises individual and group therapy based on a biopsychosocial model with emphasis on mentalization-based theory and practice [36]. An individual treatment plan, which includes treatment goals, is prepared in cooperation with each patient. Medical treatment is offered, as well as assistance/counselling for accommodation, education, employment, living, adjustment and support. Psychological treatment is tailored to the individual’s specific problems and treatment goals. The likely duration of treatment is decided with the patient as part of the treatment plan, in accordance with their needs. It can include movement between units, such as from inpatient to day patient. In the day unit, as patients become more established in school, work or a domestic situation, the therapist gradually reduces contact until discharge.

### The HAT intervention

HAT is an integral part of the department’s program of addiction treatment [37]. It comprises 12× 90-min sessions of body-orientated psychotherapy with horses. The animals have been selected and trained for this work to be strong, secure, responsive and interactive. Patients and staff are insured against injury by the hospital. Serious incidents and injuries must be recorded.

All patients are eligible to participate in HAT, but must be referred by their treating clinician. The referral can be requested by the patient or suggested by the clinician. A final decision on suitability and the treatment objectives of the individual’s HAT participation (for example, to strengthen boundary setting, or reduce anxiety, depression or aggression, etc.) are agreed at a preparatory meeting between the HAT therapist, the patient and the clinician. Patients have the opportunity to meet the horses and become involved in care activities (such as feeding) from their first day in treatment. They normally start the HAT program within 2–3 weeks. HAT therapists become part of the patient’s clinical team, with full access to the patient’s clinical record. Patients are encouraged to attend and participate fully but can choose not to undertake an activity, such as mounted work. Specific activities, level of participation and response at each HAT session are recorded by the HAT therapists in the patient’s electronic hospital journal.

The sessions are planned and provided by two qualified therapists who are also Norwegian Level 1 Riding Instructors. The program design is structured for small groups (maximum four participants per session), but includes provision for individual work on specific needs if required. It involves a three-way interactive (positive triangulated) process in which the patient works in emotional safety with the horse on activities selected with his/her therapist to address agreed goals. During sessions, the horse will respond naturally to environmental factors (for example, the proximity of other horses, or a sudden loud noise). Similarly, it will react to the physical and emotional state of the patient (for example, a request lacking focus or clarity is unlikely to produce the desired movement from the horse, and an aggressive request may be met with resistance). The therapist, in leading the process, can both read and influence the horse, and provide reflective feedback to the patient on the relationship, reactions and responses between the horse and patient.

Activities can involve any combination of herd behavior observation, stable duties, and ground, mounted and/or driving work with the horses. Observation of the herd can promote discussion of social interaction and relationships and stable duties promote responsibility, routine and reliability. Groundwork is used to address issues relating to boundaries/contact, anxiety/trust, communication/connection, mastery (of new skills, the horse and self), body awareness and focus. Mounted work addresses posture, balance/centering, coordination, rhythm/regulation, mastering of anxiety and focus. Carriage driving can be used to promote forward thinking and outlook, and, with other passengers, it can engender a sense of empowerment, group responsibility and care. These activities involve good healthy exercise, having fun, and learning new skills. However, while physical exercise, fun and skill acquisition are important, the prime purpose of this program is therapy and contribution to successful treatment.

The focus of the first four sessions is on getting to know about horses, herd behavior, basic handling and safety. The following eight sessions are tailored to meet the individual’s therapy objectives using a range of group and individual ground-based, mounted or driving exercises as outlined in the stable manual (unpublished).

The HAT program has been developed at Oslo University Hospital over time, largely by Lysell [22], a qualified and experienced body-oriented psychotherapist. It also draws on theoretical and practice material from a number of relevant equine-assisted therapy schools [16, 20, 38–42]. It uses many of the usual equine-assisted/facilitated therapy exercises but places stronger emphasis on those relevant to substance use disorders, such as boundary setting, development of trust and control of emotional affect. It differs from most other horse therapy programs in two aspects. First, the patients have responsibility for the horses after hours, giving greater emphasis on care, routine, reliability and responsibility (all relevant to substance use recovery). Second, after the four introductory sessions, the HAT program does not follow a sequenced routine. Rather, specific activities and therapeutic processing are targeted at individual patient needs and are sequenced at appropriate points throughout the patient’s HAT program.

HAT treatment outcome is not assessed per se. It is included as part of the patient’s overall treatment outcome assessment, as measured by change in the YATEP psychological instruments and, in particular, by whether individuals complete their agreed substance use treatment program.

### Statistical analysis

In this naturalistic, intention-to-treat study, univariate and multivariate analyses were used to assess the relationship between treatment completion and HAT plus a range of patient factors (gender, age, education, number and severity of substances used, psychological distress and number of temporary exits). We included time in treatment as an additional outcome measure. It is assessed using univariate analysis of both the mean (113 days) and the reported critical minimum period for effective treatment (90 days). However, we excluded time in treatment from the logistic regression because of the obvious relationship between longer time in treatment and treatment completion.

Pearson Chi squared and independent-samples *t* test were used to test the relationship between discharge status and HAT. Odds ratio (OR) was used to test the strength of the relationship. Potential confounding variables relating to the patient (age, sex, education, number and severity of substances used, and psychological distress), time in treatment (mean time and temporary exits) and HAT participation were controlled for using logistic regression analysis. Linear and other interactive associations were checked. SPSS Version 21 (IBM Corp., Armonk, NY, USA) was used.

## Results

### Study participants’ characteristics

The study involved 108 individuals: 78 males and 30 females (27.8 %). At the time of entry to treatment, 14 (13.0 %) were aged less than 20 years, 74 (68.5 %) were 20–26 years and 20 (18.5 %) were aged 27 years or older (Table 1). The mean age (results not shown) was 23.1 years (range 17–33 years, SD = 3.4). Females (mean 22.9 years, SD = 3.5) were slightly, but not significantly, younger than males. None of the patients were under legal mandate to remain in treatment.

**Table 1:** Comparison of patient characteristics: treatment as usual (not-horse assisted therapy) with intervention group (treatment as usual plus horse assisted therapy)

Variable	Item	Not-HAT^a^	HAT^a^	Total	%	Chi square
		N = 43	N = 65	N = 108	100	
Discharge status	Dropped out	37 (86.0)	28 (43.1)	65	60.2	19.94, p < 0.001
	Completed	6 (14.0)	37 (56.9)	43	39.8	
Days in treatment						
Mean	<113 days	32 (74.4)	28 (43.1)	57	55.6	10.30, p = 0.001
	113+ days	11 (25.6)	37 (56.9)	51	44.4	
Critical period	<90 days	28 (65.1)	21 (32.3)	49	45.4	11.24, p = 0.001
	90+ days	15 (34.9)	44 (67.7)	59	54.6	
Gender	Female	8 (18.6)	22 (33.8)	30	27.8	2.10, p = 0.083
	Male	35 (81.4)	43 (66.2)	78	72.2	
Age	<20 years	9 (20.9)	5 (7.7)	14	13	6.80, p = 0.034
	20–26 years	30 (69.8)	44 (67.7)	74	68.5	
	27+ years	4 (9.3)	16 (24.6)	20	18.5	
Years of schooling	<10 years	7 (16.3)	11 (16.9)	18	16.7	2.42, p = 0.298
	10–12 years	31 (72.1)	39 (60.0)	70	64.8	
	13+ years	5 (11.6)	15 (23.1)	20	18.5	
Primary substance	Cannabis	20 (46.5)	22 (33.8)	42	38.9	6.31, p = 0.389
	Alchol	6 (14.0)	14 (21.5)	20	18.5	
	Heroin	6 (14.0)	11 (16.9)	17	15.7	
	Amphetamine	4 (9.3)	11 (16.9)	15	13.9	
	Benzodiazepine	3 (7.0)	5 (7.7)	8	7.4	
	GHB^b^	2 (4.7)	2 (3.1)	4	3.7	
	Cocaine	2 (4.7)	0 (0.0)	2	1.9	
No. of substances	1 substances	12 (27.9)	23 (35.4)	35	32.4	0.82, p = 0.662
	2 substances	14 (32.6)	17 (26.2)	31	28.7	
	3 substances	17 (39.5)	25 (38.5)	42	38.9	
Age of first use	<16 years	25 (58.1)	32 (49.2)	57	52.8	0.82, p = 0.364
	16+ years	18 (41.9)	33 (50.8)	51	47.2	
HSCL-25^c^	<1.75	9 (20.9)	10 (15.4)	19	17.6	0.55, p = 0.459
	1.75+	34 (79.1)	55 (84.6)	89	82.4	
Subsitution medicine^d^	No	38 (88.4)	54 (83.1)	92	85.2	0.58, p = 0.450
	Yes	5 (11.6)	11 (16.9)	16	14.8	
Psych. co-morbidity (at entry)	None diagnosed	21 (48.8)	37 (56.9)	58	53.7	1.10, p = 0.580
	1 diagnosed	16 (37.2)	18 (27.7)	34	31.5	
	2+ diagnosed	6 (14.0)	10 (15.4)	16	14.8	
Temporary exit	No exit	32 (74.4)	33 (50.8)	65	60.2	6.10, p = 0.05
	1 re-entry	6 (14.0)	18 (27.7)	24	22.3	
	2+ re-entries	5 (11.6)	14 (21.5)	19	17.6	

^a^Not horse assisted therapy/horse assisted therapy

^b^Gamma hydroxybutyrate (C_4_H_8_O_3_)

^c^Hopkins Symptom Checklist-25

^d^Opioid pharmacotherapy

In the 6 months prior to intake, 32.4 % of the 108 patients had used a single drug, 28.7 % had used two drugs and 39.9 % had used three or more drugs. Cannabis was the most commonly used primary drug (38.9 %), followed by alcohol (18.5 %), amphetamine + cocaine (15.8 %), heroin (15.7 %) and other drugs (11.1 %). The average age of first use of the primary drug was 17 years, (results not shown) with 52.8 % of all patients being 15 years or younger when they first used their primary drug (Table 1).

At referral, all patients had a primary diagnosis of mental and behavioral disorders due to psychoactive substance use (ICD 10). Fifty-eight (53.7 %) of the patients were reported to have no psychiatric comorbid condition, 34 (31.5 %) had one, and 16 (14.8 %) had two or more. The most common of the comorbid conditions (results not shown) were behavioral disorders (36.2 %), followed by neurotic/stress (including post-traumatic stress disorder) disorders (34.8 %), mood disorders (20.3 %) and other disorders (8.7 %). Approximately 1 week after entry, 89 (82.4 %) patients’ first Hopkins Symptom Check List 25 (HSCL-25) scores were equal to or above the psychosocial risk cutoff. Sixteen patients (14.8 %) were prescribed substitution medicine (15 buprenorphine, 1 methadone) during all or part of their treatment (Table 1).

### HAT participation

Forty-three patients (39.8 %) received treatment as usual (non-HAT group) and 65 (60.2 %) had treatment as usual plus HAT (HAT intervention group). The intervention was well tolerated with no reported adverse treatment effect or injury arising from HAT. None of the patients were withdrawn from HAT for clinical reasons.

Although this was a naturalistic study, there were no major differences in baseline characteristics between the HAT group and the non-HAT group. However, significantly more HAT participants were aged 27 years or more (24.6 vs 9.3 %, p = 0.03) and their average age was slightly older than non-HAT participants (mean 23.7 years, SD = 3.4 vs mean 22.1 years, SD = 3.2, p < 0.02) (results not shown). They were also more likely than non-HAT participants to have one or more temporary exits from treatment (49.2 vs 25.6 %, p = 0.05) (Table 1).

Thirty-seven (56.9 %) of HAT participants completed their treatment compared with only six (14.0 %) of non-HAT participants, p < 0.001 (Table 1).

### Time in treatment

Days in treatment ranged from 1 to 555, (mean 113, SD = 92.7). Twenty-six (24.0 %) of the 108 patients remained in treatment for less than 30 days, 49 (21.3 %) for 30–89 days and 59 (54.6 %) for 90 days or more (results not shown). Forty-eight (44.4 %) remained in treatment for the mean 113 days or more, and 33 (76.7 %) of those completed treatment (Table 2).

**Table 2:** Comparison of discharge status: dropout or completed by patient characteristics

Variable	Item	Dropout	Completed	Total	%	Chi square
		N = 65	N = 43	N = 108	100	
Participation in HAT	No	37 (56.9)	6 (14.0)	43	39.8	19.94, p < 0.001
	Yes	28 (43.1)	37 (86.0)	65	60.2	
Days in treatment						
Mean	<113 days	50 (76.9)	10 (23.3)	60	55.6	30.18, p < 0.001
	113+ days	15 (23.1)	33 (76.7)	48	44.4	
Critical period	<90 days	44 (67.7)	5 (11.6)	49	45.4	32.82, p < 0.001
	90+ days	21 (32.3)	38 (88.4)	59	54.6	
Gender	Female	16 (24.6)	14 (32.6)	30	27.8	0.81, p = 0.367
	Male	49 (75.4)	29 (67.4)	78	72.2	
Age	<20 years	12 (18.5)	2 (4.7)	14	13	4.81, p = 0.090
	20–26 years	43 (66.2)	31 (72.3)	74	68.5	
	27+ years	10 (15.4)	10 (23.3)	20	18.5	
Years of schooling	<10 years	13 (20.0)	5 (11.6)	18	16.7	1.96, p = 0.376
	10–12 years	42 (64.6)	28 (65.1)	70	64.8	
	13+ years	10 (15.4)	10 (23.3)	20	18.5	
Primary substance	Cannabis	23 (35.4)	19 (44.2)	42	38.9	4.75, p = 0.577
	Alchol	14 (21.5)	6 (14.0)	20	18.5	
	Heroin	12 (18.5)	5 (11.6)	17	15.7	
	Amphetamine	7 (10.8)	8 (18.6)	15	13.9	
	Benzodiazepine	5 (7.7)	3 (7.0)	8	7.4	
	GHB^a^	2 (3.1)	2 (4.7)	4	3.7	
	Cocaine	2 (3.1)	0 (0.0)	2	1.9	
No. of substances	1 substances	20 (30.8)	15 (34.9)	35	32.4	0.15, p = 0.292
	2 substances	16 (24.6)	15 (34.9)	31	28.7	
	3 substances	29 (44.6)	13 (30.2)	42	38.9	
Age of first use	<16 years	35 (53.8)	22 (51.2)	57	52.8	0.08, p = 0.785
	16+ years	30 (46.2)	21 (48.8)	51	47.2	
Substitution medicine^b^	No	55 (84.6)	37 (86.0)	92	85.2	0.42, p = 0.840
	Yes	10 (15.4)	6 (14.0)	16	14.8	
HSCL-25^c^	<1.75	12 (18.5)	7 (16.3)	19	17.6	0.09, p = 0.771
	1.75+	53 (81.5)	36 (83.7)	89	82.4	
Psych co-morbidity (at entry)	None diagnosed	32 (49.2)	26 (60.5)	58	53.7	2.25, p = 0.325
	1 diagnosed	24 (36.9)	10 (23.3)	34	31.5	
	2+ diagnosed	9 (13.8)	7 (16.3)	16	14.8	
Temporary exit	No exit	42 (64.6)	23 (53.5)	65	60.2	1.87, p = 0.393
	1 re-entry	14 (21.5)	10 (23.3)	24	22.3	
	2+ re-entries	9 (13.8)	10 (23.3)	19	17.6	

^a^Gamma hydroxybutyrate (C_4_H_8_O_3_)

^b^Opioid pharmacotherapy

^c^Hopkins Symptom Checklist-25

HAT participants remained in treatment for a significantly longer period (p < 0.001) than those who did not participate in HAT (mean 141 days, SD = 93.6, vs mean 70 days, SD = 73.8). They were almost four times more likely to remain in treatment for 90 days or more (OR 3.9 CI 1.7–8.8, p = 0.001) (results not shown).

### Treatment discharge status

At discharge, 43 (39.8 %) of the 108 patients in the study had completed treatment and 65 (60.3 %) had dropped out. Treatment completion was significantly associated with only HAT participation [χ^2^ (1,108) = 19.9, p < 0.001] and length of time in treatment {for both mean days in treatment [χ^2^ (1,108) = 30.2, p < 0.001] and the critical 90 days period [χ^2^ (1,108) = 32.8, p < 0.001] (Table 2)}.

### Prediction of treatment completion

Apart from the length of time in treatment, HAT participation was the only significant univariate predictor of treatment completion (OR 8.2, CI 3.0–22.0, p < 0.001) (results not shown). Excluding time in treatment, and after controlling for the potentially confounding influence of age, sex, education, number and severity of substances used, psychological distress and temporary exits, the adjusted odds ratio for HAT participants completing treatment was 8.4 (95 % CI 2.7–26.4, p < 0.001), (Table 3).

**Table 3:** Logistic regression predicting likelihood of completing treatment: a multivatiate model of all variables listed

Variable	Item	Total	%	p value	Odds ratio	Confid. lower	Interval upper
Participation in HAT	No (0)	43	39.8				
	Yes (1)	65	60.2	<0.01	8.42	2.7	26.4
Gender	Female (0)	30	27.8				
	Male (1)	78	72.2	0.84	0.90	0.3	2.6
Age	<20 years (0)	14	13	0.25			
	20–26 years (1)	74	68.5	0.11	4.37	0.7	26.0
	27+ years (2)	20	18.5	0.13	4.78	0.6	36.2
Years of schooling	<10 years (0)	18	16.7	0.41			
	10–12 years (1)	70	64.8	0.20	2.58	0.6	10.1
	13+ years (2)	20	18.5	0.30	2.40	0.5	11.3
No. of substances	3 substances (0)	42	38.9	0.25			
	2 substances (1)	31	28.7	0.09	2.66	0.9	8.4
	1 substances (2)	35	32.4	0.43	1.60	0.5	5.1
Substance severity^a^	Less severe (0)	62	57.4				
	More severe (1)	46	42.6	0.47	1.42	0.6	3.7
HSCL-25^b^	<1.75 (0)	19	17.6				
	1.75+ (1)	89	82.4	0.90	1.11	0.3	3.9
Temporary exit	2+ re-entries (0)	19	17.6	0.67			
	1 re-entry (1)	24	22.3	0.37	0.53	0.1	2.1
	No exit (2)	65	60.2	0.58	0.70	0.2	2.5

^a^Less severe (cannabis and alchol) more severe (heroin, amphetamine, benzodiazepine, Gamma hydroxybutyrate, cocaine)

^b^Hopkins Symptom Checklist

## Discussion

This study found a statistically significant association between HAT participation and longer time in treatment, and between HAT participation and completion of treatment. Although not direct measures of substance use, both duration and completion of treatment are reported in previous studies to be predictors of positive treatment outcome for substance use disorders [3, 4, 8, 9].

Length of time in treatment was the strongest predictor of treatment completion. This is consistent with previously reported studies [1, 3, 13], but it may hide the influence of other patient or treatment variables [4, 10], such as HAT in our study. The optimal duration of treatment is debatable and may depend upon the individual needs of the patient and the type of treatment method [10]. However, 90 days is often identified as the minimum period for effective treatment [2, 3, 11–13].

HAT participants were significantly more likely than non-HAT participants to remain in treatment for 90 days or more and to complete their treatment. However, they were also more likely to have had a temporary exit. This may seem anomalous with some regarding any unscheduled exit as negative. However, return to complete treatment is encouraged and it is possible that attachment to the horses may have made the decision to return a little easier for some. Further controlled studies are needed to clarify the causal relationship between HAT and temporary exit, duration and completion of treatment.

A major challenge in addiction treatment is to identify what treatment modality or other factors motivate patients to stay in treatment [6, 10, 43, 44]. Treatment factors (method, setting, duration, staff/patient ratio) and treatment process factors (motivation, alliance, satisfaction, and interaction) are amongst the best predictors of treatment outcome [6, 44]. Furthermore, Simpson and colleagues suggest that use of therapeutic process and environmental influences as treatment enhancement may improve substance use outcomes [45]. HAT, in this study, is an innovative adjunct-treatment factor.

From the substance use and the HAT literature, we identified a number of possible explanations for why HAT participants may remain in and complete treatment. These include therapeutic alliance, the environment, physical activity, staff influence, individual attention and comorbidity.

Relational factors and therapeutic alliance can predict retention in treatment [1] and treatment outcome [44]. Premature termination of treatment and poor therapeutic alliance have been consistently reported in the scientific literature as predictors of negative treatment outcome [2–4, 6, 46]. Many HAT studies refer to the human–horse relationship as a positive motivating factor in therapy [16, 18, 20, 31, 47]. Burgon argues that building rapport with the horse increases comfort in the therapeutic relationship and leads to positive change and learning [25]. This may well be true, but we were unable to find substantiating quantitative studies of HAT and therapeutic alliance. It warrants further study.

As other experienced therapists report, many young patients respond better to the therapist in an active, less verbal environment than they do sitting in the more formal environment of a therapist’s office [20, 43, 46, 47, 53]. Inclusion of an adjunct activity, such as gardening, music or art therapy, is also reported to be associated with successful treatment completion, as Decker et al. found in their pilot study of novel treatments [43].

A pleasant environment, physical activity and hobbies can have a beneficial impact in addiction and related psychological treatment [48–52]. HAT is a body-oriented psychotherapy with a range of physical activities, most of which occur outdoors in a pleasant, natural and quiet environment where therapeutic activities can be adapted to the seasons. Staff can also influence retention [2, 4, 5]. The HAT team works in a relaxed, non-judgmental atmosphere with patients, to explore and work on their issues.

Patients “are likely to continue in treatment longer … with individual attention and when seen in smaller groups in friendly comfortable environments” (Stark, 1992, p. 93) [3]. HAT sessions are conducted in small groups of not more than four patients with four staff, or in individual patient–therapist sessions. Patients can choose to work with their preferred horse, and most have a favorite. Other substance use studies have found focused personal attention, to be a key element in participants’ retention in treatment [13, 43]. It is possible that the focused horse and human attention during HAT is a contributing factor to patients’ retention in treatment.

Depression, anxiety, aggression, poor motivation and low self-esteem are among the most commonly cited psychological conditions associated with and responding to therapy with horses [16, 18, 21]. These conditions are also common elements of addiction comorbidity [54, 55]. The literature shows that distress scores on the HSCL-25 are very high at entry, because of the unstable condition at entry both emotionally (starting treatment) and because of the former drug use lifestyle. This may be one reason why we found no association between HSCL-25 and completion of treatment. Distress scores should be re-examined in a broader study of HAT and comorbidity.

Given the naturalistic study design, we cannot infer causality. Nor should the results be interpreted as an assessment of HAT as a therapeutic process (for substance use or for other psychological disorders). The purpose of the study was simply to examine whether, as some patients claimed, participation in the HAT program was associated with longer duration and completion of treatment. Further work is required to understand better HAT’s therapeutic processes, and the underlying causes of, and impact on comorbidity and longer-term treatment effect.

### Strengths and limitations

The size and relative homogeneity of the population studied is a strength when compared with other studies of therapy involving horses. However, there are limitations to both internal and external validity. Nonrandom assignment, including possible self-selection bias, is the most obvious limitation. The possible novelty effect of HAT is another. These shortcomings need to be addressed in large controlled studies that seek to clarify HAT’s role, including causal inference, in substance use disorder therapy, in treatment outcomes and treatment effect.

HAT participation was voluntary. Because there were no data on why patients joined or did not join the HAT program, it was not possible to form an opinion on whether the HAT participants stayed in treatment longer because they participated in the HAT program or because of some other positive factor, which also led them to participate in HAT. Data on patients’ motivation and program satisfaction (such as those used by Decker et al. [43]) might have been useful but were not available.

Dropout, the most frequently used measure of addiction treatment outcome, is fraught with definitional and other problems [3, 4, 6, 10]. There is no consistent definition of the term. Many studies fail to define how they have used it and/or omit discussion of the impact of temporary exits. We did not find any studies that included dropout as a measure of treatment outcome for HAT, and therefore comparisons were not possible.

Our findings are consistent with claims that HAT has a positive effect on psychosocial illness. However, we acknowledge this could be due, in whole or in part, to a variety of possible confounding factors that were not examined (such as, for example the “honeymoon” effect of a new program, the outdoor environment or the ambience around the stables). These factors lend themselves initially to qualitative investigation.

It is possible that a similar effect might be obtained more economically with another animal, such as a dog. Inclusion of dogs in substance misuse therapy has been found to improve therapeutic relationships [56]. However, we have found no studies which compare the use of horses with other animals as an adjunct therapy for substance misuse but note that Nurenberg [32] and colleagues in their comparison did find a significantly positive effect of therapy involving horses, but not dogs, when incorporated into psychotherapy for aggressive behavior.

### Relevance

Ongoing care was not addressed in the study. However, HAT may have considerable potential as a community-based activity for outpatient treatment. If available from accredited public and private providers in community settings, it may enable engagement in healthy, pleasurable outdoor activities with a nonsubstance use peer group.

There is a continuous struggle to find what motivates substance use disorder patients to remain healthy. Rigorous evidence of safety and efficacy is required before HAT can become a conventionally accepted treatment with the associated provision of health insurance cover [57–59]. The positive findings from this treatment facility-based study, and HAT’s broader community potential, prompt further investigation of both the underlying therapeutic processes and the longer-term impact of HAT on substance use disorder treatment.

## Conclusion

This naturalistic study used intention-to-treat analysis to examine HAT in a substance use disorder treatment program for young adults. The objective was to assess whether HAT patients remained in treatment longer and were more likely to complete their agreed program of treatment than were non-HAT patients. It found statistically significant associations between HAT participation and duration and completion of treatment. These findings are consistent with the claims of patients and the growing body of equine therapy literature. However, the study does not indicate whether either HAT or the horses per se might have accounted for the results. Investment is needed in larger controlled studies, in qualitative investigation of the patients’ and clinicians’ perspectives, and in the ethological study of the horses’ actual contribution in the therapeutic process.
